# EVERYDAY ETHICS AND THE UNSEEN MORAL LANDSCAPE OF ASSISTED LIVING

**DOI:** 10.1093/geroni/igz038.3443

**Published:** 2019-11-08

**Authors:** Candace L Kemp, Jason Lesandrini, Clifvette Webb, Elisabeth O Burgess, Jennifer C Morgan

**Affiliations:** 1 Georgia State University, Atlanta, Georgia, United States; 2 WellStar Health System, Atlanta, Georgia, United States; 3 Gerontology Institute, Georgia State University, Atlanta, Georgia, United States

## Abstract

Assisted living (AL) communities are increasingly popular long-term care settings where people live, work and visit, and where social relationships and care, including end-of-life care, are negotiated. In the context of daily life, residents, their family members and friends, AL care workers and other staff, external care providers, and volunteers, continually encounter one another and make choices about a range of matters. AL is fraught with uncertainty and conflict about values, especially given residents’ cognitive and physical frailty. These value-laden issues have important implications for both resident and care partners’ quality of life. Yet, almost no research has examined ethics in this dynamic and complex care environment. We seek to address this important knowledge gap by examining AL through an ethical lens. Drawing on our research and practice experiences, we present a conceptual model that situates everyday ethics within multiple multi-levels of moral decision-making factors and systems involving individuals, the care setting and surrounding community, social norms, and the broader regulatory context. We provide an overview of AL’s moral landscape and present examples of three everyday ethical issues. We first examine informed consent as it pertains to sexual encounters involving residents with dementia. Next, we consider boundary and role issues inherent in care process and relationships. Finally, we discuss resident privacy as an area for value uncertainty in everyday life within AL. We conclude by emphasizing the need for in-depth and systematic identification of ethical issues in AL and development of management strategies for applied practice.

